# Risk factors associated with under nutrition among children aged 6-59 months in Ngorongoro, Arusha region, Tanzania: a case-control study, 2017

**DOI:** 10.11604/pamj.2020.37.315.21726

**Published:** 2020-12-04

**Authors:** Ester Lucas Mdimu, Julius John Massaga, Senga Lucy Sembuche, Ahmed Mohamed Abade, Germana Henry Leyna

**Affiliations:** 1 Department of Epidemiology and Biostatistics, Muhimbili University of Health and Allied Sciences, P.O. BOX 65015, Dar-es-Salaam, Tanzania,; 2 Tanzania Field Epidemiology and Laboratory Training Programme, Tanzania Ministry of Health, Community Development, Gender, Elderly and Children, P.O. BOX 743-40478, Dodoma, Tanzania,; 3 National Institute for Medical Research, P.O. BOX, 9653, Dar es Salaam, Tanzania

**Keywords:** Undernutrition, children, under-five, Ngorongoro, factors, Tanzania

## Abstract

**Introduction:**

childhood undernutrition is one of the leading causes of morbidity and mortality in children below five years of age especially in developing countries like Tanzania, particularly in rural area. Inappropriate child caring and feeding practices have been strongly associated with it. Many actions have been taken to reduce prevalence of undernutrition in children in Ngorongoro district, however, the problem persist. This study identified risk factors associated with undernutrition in children under-five years of age in Ngorongoro district, Arusha region.

**Methods:**

a health facility-based unmatched case-control study was conducted with 400 (100 cases and 300 controls) children aged 6-59 months. Cases included children with undernutrition according to the WHO anthropometric indicators. Controls were children within the normal range of these indicators. Both cases and controls, were obtained from children attending the Reproductive and Child Health (RCH) for assessment of growth and development or admitted in pediatric ward. A questionnaire was used to collect demographic characteristics, child health and caring practices and environmental factors. Height, weight and Mid Upper Arm Circumference (MUAC) were measured using standard approaches. We employed logistic regression analysis to identify significant risk factors for undernutrition.

**Results:**

undernutrition was associated with young age of mothers/caregivers (adjusted Odds Ratio (aOR)=38.8, 95% CI: 15.38-59.03.); early age of initiation complementary foods (aOR=13.6, 95%CI: 3.15-59.06); a child having diarrhoea in past one month (aOR=4.0; 95%CI: 1.76-12.85); large family size (aOR=6.1, 95% CI: 2.16-16.90); low frequency of feeding (aOR=3.9, 95%CI: 1.59-9.58); low birth weight (aOR:=7.3, 95%: CI: 1.15-46.70); and source of drinking water; well (aOR=16.3, 95%: CI: 1.81-147.05) and surface water (aOR=16.18, 95%CI: 1.85-141.71).

**Conclusion:**

household and individual characteristics of the children and mother/caregiver are important predictors of undernutrition in this community. Tailored interventions, instead of blanket approaches, should be designed to mitigate and eliminate childhood undernutrition in Ngorongoro.

## Introduction

Undernutrition is one of the leading causes of morbidity and mortality in children below five years of age, particularly in developing countries [1, 2]. It consists of stunting growth which result from chronic protein deficiencies; wasting which occurred due to chronic illness during childhood; and underweight which is usually the result of inadequate balanced diet [1, 3]. Children under-five years of age are the most vulnerable because of their high nutritional requirements for growth and development as well as immunity building [1, 3, 4]. Globally in 2015, about 162 million children under-five years of age were malnourished as well as 35% of children under-five years of age who die each year. These deaths are attributed to undernutrition and the most affected were the developing countries [1, 4, 5]. In Africa alone, it was estimated that in the period from 2015 to 2016 around 39% of children under five-years were stunted, 28% were wasted and 3% were underweight [3, 5]. In Kenya, from 2015 to 2016, there was an estimate of approximate 31% stunted, 22% underweight and 8% wasted children. During the same period in Nigeria, 40% were estimated stunted, 25% underweight and 9% wasted [6].

The situation in Tanzania is not very different from other African countries; within 2015 to 2016 it was estimated that, about 42% of children were stunted, 16% were underweight and 5% were wasted [2, 7]. However, there are lot of variations from one region to another. Undernutrition was 36% in Arusha while in Morogoro and Simiyu was 33%, Lindi 35% and 39% in the Tanga region [2, 8]. The problem varies highly from one district to another even within the same region. In the Arusha region, the district that leads for reported cases of undernutrition for under-five children was Ngorongoro. Unpublished data from the District Health Information System (DHIS2) shows an increase in the prevalence of undernutrition in children under-five years in Ngorongoro district from 2016 to 2017. Factors for this increase have not yet been established.

Studies have shown that stunted children have up to a nine-fold increased risk of dying compared to well-nourished children [2, 9]. The problem decreases mental development resulting in inadequate cognitive achievement [4, 10]. A number of risk factors have been linked, most are related to inadequate diet, inadequate water supply, poor hygiene and sanitation practices [8, 11]. Other risk factors also include poor maternal nutrition and low birth weight. However, magnitude of these risk factors varies from one locality to another [9, 12]. Using an unmatched case-control study, we aimed to determine the risk factors associated with undernutrition among children under-five years of age living in Ngorongoro district.

## Methods

**Study setting:** the study was conducted in Waso District Hospital in Ngorongoro district, Arusha Region in Tanzania. Waso hospital is a referral district hospital. The district have three divisions, twenty wards and seventy two villages [13]. The district has an area of 14,036 square kilometres with an estimated population of 174,278 as projected from the 2012 National census [8, 13]. The district has a total of 22 health facilities, 2 hospitals, 3 health centres, and 17 dispensaries. The average travelling distance to a health facility is 22km, range: 0-162km [13]. The main tribe are the Maasai whose main economic activity is animal keeping (pastoralism), during dry season men leave women on their own while looking for grazing.

**Study design and study populations:** an unmatched case-control study was conducted among children under five years of age (6-59 months) residing in Ngorongoro district. Residence was defined as having a permanent address in the district and lived in district at least for the past three months preceding data collection. A Case was defined as any child between 6-59 months old presenting with any of the following World Health Organization anthropometric indicators for under nutrition-weight-for-height <-2SD or weight-for-age <-2SD or height-for-age <-2SD or measurement of Mid Upper Arm Circumference (MUAC) <12cm. Controls were defined as children between 6-59 months old considered to have normal anthropometric indicators.

**Sample size and sampling:** the sample size was computed using Epi info Statcalc 7 software for case control study following Kelsey formula of 2007 [14]. Statistical assumptions were based on a two-sided significance level of 95%; 80% power; a case to control ratio of 1:3; design effect of 2; proportion of controls exposed of 41.5%; and proportion of cases exposed of 58.7% [14, 15]. After accounting for a 5% possible non-response, the minimum sample size was 400 (100-cases and 300 controls). All cases admitted to the pediatric ward for different diagnosis or attend to Reproductive and Child Health (RCH) clinic for routing growth and development assessment between March and April 2017 were included in the study. Controls were selected in the same hospital from the pediatrics ward and RCH clinic where cases were recruited.

**Data collection instruments:** questionnaires were developed with most questions adopted from the Demographic and Health Survey (DHS) tool to capture information on demographic characteristics of the child, socio-economic and demographic characteristics of the mother/caregiver, child health and caring practices and environmental factors. The questionnaire was developed in the English language and later translated to Swahili. Research assistants were trained for two days at Waso Hospital hall on measuring the children weigh, height, age and to fill growth and development card. The research assistance was also required to know Kiswahili and Maasai for easy communication.

**Measurements:** anthropometric and MUAC measurements were taken from every child attending the RCH for assess growth and development and admitted to the paediatric ward for treatment of different diagnosis following standard WHO procedures. In brief, weight was measured with minimal clothing and recorded to the nearest 0.1kg using Salter scale (Zhezhong, China). Height was measured to the nearest 0.1cm with the child in the upward upright position, with legs stretched to a full extent and feet at right angles with legs and for those who could not stand recumbent length of the child on a measuring board was measured. The child´s mid-arm circumference was measured to the nearest 0.1cm using a non-stretchable tape placed around the upper arm at the midway point. Care was taken to make sure that the tape fitted comfortably around the child´s arm.

**Data analysis:** data was entered, cleaned and analyzed using Epi info version 3.1.5. Undernutrition frequency distribution tables were constructed. The outcome variable undernutrition in children under-five years was associated with individual predictor variables (age of the mother/caregiver, age initiation of feeding, low birth weight, diarrhoea disease in the past on month, home delivered, family with seven people in the house, marital status of the mother/caregiver, level of education of the mother/caregiver, occupation ). In this comparison the measure of association (odds ratio) and its statistical significance were estimated using the Chi-squared test at the significant level of 5%. Multivariable logistic regression was performed to identify the significant factors contributing to undernutrition while controlling for other factors. During the stepwise modelling regression analyses, all variables with a p≤0.1 in the bivariate analysis were considered for inclusion.

**Ethical consideration:** ethical approval was obtained from the senate of Muhimbili University of Health and Allied Sciences Research and Publication Committee with the following reference number MU/PGS/SAEC/Vol.XVIII. Permission to conduct the study and work with communities was obtained from all level of administration including the Arusha region, Ngorongoro district, village authorities and local tribal leaders. Participation in this study was voluntary so participants had benefits to get knowledge and asking any question pertaining to study. Participants had right to withdraw from the study at his/her will without any penalty and written or thumb print informed consent was sought from all participants. Confidentiality was maintained in the study by assuring that all questionnaires collected did not identify the participants in any way and the recorded information were kept together by using numbers.

## Results

**Socio-demography of mothers and their children recruited for the study in the Ngorongoro district Tanzania 2017:** a total of 400 children participated in this study, about 49% of cases and controls were boys while 51% of cases and controls were girls. Cases (46%) and controls (43%) were children between 12-23 months. The median age for cases was 19 months (range: 14-30.5 months) while the median age for controls was 23 months (range: 15-33 months). IQR for cases was 22 months (14-30.5 months) and a control was 24 months (15-33 months). A significantly smaller proportion of cases were born ≥2.5kgs than controls (cases 71.9% versus controls 95.3%; p<0.001). Forty two percent of cases had 3 siblings who were under-five compared to 6.7% for controls (p<0.001) (Table 1).

**Table 1:** socio-demographic of mothers and their children recruited in the study, Ngorongoro district Tanzania, 2017

Characteristics	Cases (n=100) n (%)	Controls (n=300) n (%)	P value
**Sex of the child**			
Male	49 (49.0)	147 (49.0)	
Female	51 (51.0)	153 (51.0)	1.0
**Age of child in months**			
6-11	20 (20.0)	27 (9.0)	
12-23	46 (46.0)	129 (43.0)	
12-23	16 (16.0)	76 (25.3)	
36-47	14 (14.0)	57 (57.0)	
48-59	4 (4.0)	11 (3.7)	0.02
**Child´s birth weight (born at hospital)**			
<2.5	9 (28.1)	8 (4.7)	
≥2.5	23 (71.9)	164 (95.3)	< 0.001
**Number of under five children in house hold**			
1	4 (4.0)	96 (32.0)	
2	54 (54.0)	184 (61.3)	
3	42 (42.0)	20 (6.7)	< 0.001
**Child lives with**			
Mother and father	95 (95.0)	262 (87.3)	
Mother only	5 (5.0)	38(12.7)	0.03
**Age of mother/caregiver years**			
17-22	71 (71.0)	26 (8.7)	
23-28	26 (26.0)	148 (49.3)	
29+	3 (3.0)	126 (42.0)	< 0.001
**Husband with two or more other wives**			
Yes	57 (57.0)	165 (55.0)	
No	43 (43.0)	135 (45.0)	0.73
**Mother/caregiver education**			
None	45 (45.0)	118 (39.3)	
Primary	52 (52.0)	117 (42.3)	
Secondary and Above	3 (3.0)	65 (21.7)	0.5
**Father education**			
None	37 (37.0)	49 (16.3)	
Primary	53 (53.0)	148 (49.3)	
Secondary and Above	10 (10.0)	103 (34.3)	0.5
**Mother/caregiver occupation**			
Pastoralist	66 (66.0)	147 (49.0)	
Peasant	23 (23.0)	41 (13.7)	
Others	11 (11.0)	112 (37.3)	0.9
**Father occupation**			
Pastoralist	65 (65.0)	76 (25.3)	
Peasant	13 (13.0)	40(13.3)	
Others	22 (22.0)	184 (61.4)	0.5

**Child health, caring practice, environmental factors and their association with undernutrition in children 2017:** most of the cases were delivered at home than controls (69% vs. 47%; p<0.001). About 50% of cases and controls were still breastfeeding during the study. More cases were weaned ≤12 months compared to controls (46% cases versus 11.7% controls). More cases significantly reported suffering from pneumonia (56%), diarrhoea (82%) and urinary tract infection (23%) in the past one month compared to 37%, 41% and 14% of controls, respectively. Fewer cases (79%) reported being dewormed over the last three months compared to 92% controls (p<0.001). A family size <7 were reported by 11 (11%) for cases and 177 (59%) in controls while 89% of cases lived in households with 7 or more members compared to 41% controls (p<0.001). Majority of cases (66%) reported using surface water (such as streams, rivers) as a source of drinking water compared to 40% controls (p<0.001). More mothers/caregivers of cases (72%) reported taking one hour or more to fetch water compared to controls (32.3%). Less cases reported to treat water to make it safe as compared to controls (22% versus 52.3%; p<0.001). Only a third (35%) of mothers/caregivers of cases reported having latrine in household compared to 61.7% of controls (p<0.001) (Table 2).

**Table 2:** child health, caring practice and environmental factors and their association with under-nutrition in children, 2017

Characteristics	Case (n=100) n (%)	Control (n=300) n (%)	P value
**Place of birth**			
Home	69 (69.0)	141 (47.0)	
Hospital	31 (31.0)	159 (53.0)	0.0014
**Age of initiation complementary feeding (months)**			
<4	93 (93.0)	151 (50.3)	
≥4	7 (7.0)	149 (49.7)	<0.001
**Weaning age**			
≤12	23 (46.0)	18 (11.7)	
13-23	21 (42.0)	58 (37.7)	
≥24	6 (12.0)	78 (50.6)	<0.001
**Frequency of feeding**			
<6 times per day	71 (71.0)	55 (18.3)	
≥6 times per day	29 (29.0)	245(81.7)	<0.001
**Illness in the past one month (each illness tested individually)**			
Malaria (% Yes)	8 (8.0)	17 (5.7)	
Pneumonia (%Yes)	56 (56.0)	112 (37.3)	
Diarrhoea (%Yes)	82 (82.0)	123 (41)	
Urinary tract infection (%Yes)	23 (23.0)	43 (14.3)	0.04
**Deworming of the child in last 3 months (%Yes)**	79 (79.0)	277 (92.3)	<0.001
**Family size**			
<7 People	11(11.0)	177 (59.0)	
≥7 People	89 (89.0)	123 (41.0)	<0.001
**Source of drinking water**			
Well	33 (33.0)	72 (24.0)	
Surface water	66 (66.0)	120 (40.0)	
Pipe water	1 (1.0)	108 (36.0)	<0.001
**Time for searching water (in minutes)**			
1-30	4 (4.0)	126 (42.0)	
31-59	24 (24.0)	77 (25.7)	
60+	72 (72.0)	97 (32.3)	<0.001
**Treatment of drinking water**			
Yes	22 (22.0)	157 (52.3)	
No	78 (78.0)	143 (47.7)	<0.001
**Availability of a latrine in household**			
Yes	35 (35.0)	185 (61.7)	
No	65 (65.0)	115 (38.3)	<0.001

Multivariable logistic regression analysis of undernutrition showed that socio-demographic characteristics of child and household, child health and caring practices as well as environmental factors were significantly associated with undernutrition. Mothers/caregivers <25 years were 38 times more likely to have a child with undernutrition than mothers who were 25 years or older (aOR=38.8; 95% CI: 15.38-98.03, p<0.001). The families with ≥7 people were six times more likely to have a child with undernutrition compared to families with <7 people (aOR=6.1:95% CI 2.16-16.90, p<0.01). Children who were initiated on complimentary foods when <4 months were around fourteen times more likely to have undernutrition compared to children four months or older (aOR=13.6; 95% CI: 3.15-59.06, p<0.01). Children who were feed <6 times per day had a four fold probability of having undernutrition than children feed ≥6 times per day (aOR=3.9, 95% CI; 1.59-9.58; p<0.01). Children born <2.5kg were seven times more likely to have undernutrition compared to children born ≥2.5kg (aOR=7.3:95% CI: 1.56-46.70, p<0.02). Children who had diarrhoea in the past one month were five times more likely to have undernutrition than children who did not report having diarrhoea in the past one month (aOR=4.8;95% CI:1.76-12.85, p< 0.01). Families that reported using a well as a source of drinking water (aOR=16.3; 95% CI: 1.81-147.05, p<0.02) or using surface water for drinking (aOR=16.2; 95% CI: 1.85-141.71; p<0.02) were also significantly associated with undernutrition (Table 3).

**Table 3:** factors associated with under nutrition among under-five children in Ngorongoro District 2017

Characteristics	Crude OR (95%CI)	Adjusted OR (95%CI
**Age of mother/caregiver**		
<25 years	28.2** (14.48-54.73)	38.1**(15.38-98.03)
≥25 years	1	1
**Age of initiated complementary feeding**		
<4 months	13.1** (5.89- 29.20)	13.6** (3.15-59.06)
≥4 months	1	1
**Child who had diarrhoea in the past one month**		
Yes	6.6** (3.75-11.47)	4.8** (1.76-12.85)
No	1	1
**Family size**		
≥7 people	11.6** (5.97-22.69)	6.1** (2.16-16.90)
<7 people	1	1
**Frequency of feeding**		
<6 times per day	10.9**(6.47-18.37)	3.9*(1.59-9.58)
≥6 times per day	1	1
**Birth weight**		
<2.5kgs	3.6*(1.35-9.63)	7.3*** (1.15- 46.70 )
≥2.5kgs	1	1
**Source of drinking water**		
Well	49.5** (6.62- 370.03)	16.3* (1.81-147.05)
Pipe	1	1
Surface (river, lake, dam)	59.4**(8.11-435.31)	16.2*(1.85-141.71)
Pipe	1	1

OR = Odds ratio, C.I. = Coefficient interval, * =p< 0.01, ** = p< 0.001, *** = p < 0.05

## Discussion

In the study, we found that undernourished children were more likely to have a young mother/caregiver than normally nourished children this could be probably due to young having a mother/caregiver who had inadequate knowledge on feeding practice and child care experience. These findings were in line with findings reported in similar studies of under-nutrition conducted in Kenya and India [16, 17]. The study found that, children with low birth weight were more likely to be undernourished as compared to those born with normal weight. This could be due to the fact that children born with low weight continues to grow slowly due to impact of nutrition in the first year of life compared to the children born with normal weight, this result is similar to the findings reported in Zimbabwe, Nigeria and Congo [17, 18]. Large family sizes which included seven or more people, increased a risk of having an under-nourished child. This could be due to increased sharing of limited resources in a household including food which may result to inadequate food intake for the under-five children and accessibility of health care, economic status and sometime crowding can lead to infectious disease transmission. The findings were similar to results reported in Ethiopia, Malawi and Iran [11, 19].

This study found that initiation of a complementary diet before four months of age was significantly associated with under nutrition among the study population. This perhaps was due to the fact that, they believe complementary food is better for babies. These results were similar to other studies done in Zimbabwe, Tanzania, and Uganda [2, 17, 20]. The study also observed that children who had diarrhoea in the past one month before data collection, were more likely to be immediate wasting as compared to children who didn´t have diarrhoea disease in the past one month. This finding is possibly related to the source of the water used in the households. Similar findings were reported in Mozambique and Ethiopia [21, 22]. Families that used well and surface water (dam, river) as source of drinking water were more likely to have children with under nutrition as compared to families that used piped water. Drinking untreated well and surface water exposes the users to pathogens due to the higher possibility of contamination with human and animal faeces, leading to diarrhoea which ultimately results in under nutrition among children. Frequent illnesses, particularly, diarrhea, have been reported in other sub-Saharan Africa (SSA) countries to be among the core factors associated with poor nutrition in children [23-25].

This is an analytical study so is able to infer causality between studied factors and undernutrition. However, our results should be considered with the following limitation. Recall bias likely to be non-differential as both cases and controls were admitted or visiting the health facility for a health reason. The effect of this bias would thus underestimate the association observed. However, question were arranged to stimulate recall and the reference time was reduced to five years. Also, selection (Berksonian) bias as we recruited cases and controls that visited the hospital which is not representative of the community dwelling children.

## Conclusion

Under nutrition among children in Ngorongoro, Arusha region, Tanzania 2017 was a health problem for under five children, the intervention should focus on strengthening Reproductive and Child Health (RCH) on going education concerning feeding practice to under five as well as caring the children under five.

### What is known about this topic

- Age of mother/caregiver, below 25 years significantly associated with undernutrition in children under-five years of age;
- Child birth weight below 2.5kg, significantly associated with undernutrition in children under-five years of age;
- Family sizes above seven people in the house as well as surface as source of water were determinants of undernutrition in children under-five years of age in Ngorongoro district.

### What this study adds

- Children who were feed less than six per day increase the odds of undernutrition around four times as much as those feed at six or more per day. In this study suggests that every mother/caregiver should be teach on under-five children feeding practice during delivery, RCH clinic and pediatric wards and awareness programs on affordable nutritious foods that are locally available should be introduced by the local government through community participation;
- Children who had diarrhoea in the past one month were about five times more likely to have undernutrition than children who did not report having diarrhoea in the past one month. In this study recommend that proper seeking of hospital advice and care during illness should be made to the mothers/care give during RCH clinic as well as in the community at larger.
